# Gain related to the care of people with dementia: concept analysis

**DOI:** 10.15649/cuidarte.5112

**Published:** 2026-04-28

**Authors:** Alba N Lozano-Romero, Claudia Miranda-Castillo

**Affiliations:** 1 Nurse, Master of Science in Nursing, Assistant Professor, Department of Nursing, Faculty of Medicine, University of Chile. Doctoral student in nursing science, Andrés Bello University, Postgraduate student at the Millennium Institute for Nursing Research (MICARE), Chile. Santiago, Chile. E-mail: alba.lozano@uchile.cl University of Chile Santiago Chile alba.lozano@uchile.cl; 2 Psychologist, PhD in Aging and Mental Health, UCL. Director of the Millennium Institute for Care Research (MICARE). Associate Professor, Faculty of Nursing, Andrés Bello University. Millennium Institute for Research on Depression and Personality, Santiago, Chile E-mail: claudia.miranda@unab.cl Andrés Bello University Santiago Chile claudia.miranda@unab.cl

**Keywords:** Dementia, Reward, Outcome Expectations, Caregivers, Demencia, Recompensa, Expectativas de Resultados, Cuidadores, Demência, Recompensa, Expectativas de Desfechos, Cuidadores

## Abstract

**Introduction:**

The concept of gain of caring has been used as a synonym for positive aspects of care, making it difficult to use in research and practice.

**Objective:**

To clarify the concept of care gain in dementia contexts.

**Materials and Methods:**

The analysis of the concept of gaining care was conducted according to Walker and Avant's Methodology in 2019, with the seven steps adjusted for this study. Data were collected by a literature search during August 2024, in the databases SCOPUS, WEB OF SCIENCE, CINAHL, ProQuest, LILACS, and a manual search of article references with no time limit. Eighteen original and review studies were included in the analysis.

**Results:**

The conceptual clarification defines caregiving gain as a positive, enriching consequence that emerges from the interaction among contextual components (individual, relational, community, and social), as well as the quality and quantity of direct care activities and the duration of the caregiving role. This gain transcends both the active caregiving period and the stage after its completion.

**Discussion:**

The gain of caring includes contextual elements, time, and care burden variables, whose interactions enrich the caregiver experience and enhance the quality of care. This clarification, while recognizing its phenomenological nature, will facilitate its appropriate use in research and practice of dementia caregiving.

**Conclusion:**

In family caregiving in dementia, the gain of care is an enriching consequence for the caregiver and improves the quality of care. It is suggested that its subjective nature be considered in future studies and nursing practice.

## Resumen

**Introducción:**

El concepto de ganancia del cuidado ha sido usado como sinónimo de aspectos positivos del cuidado, generando dificultad para su uso investigativo y práctico.

**Objetivo:**

Clarificar el concepto de ganancia del cuidado en contextos de demencia.

**Materiales y métodos:**

Este análisis de concepto utilizó la Metodología de Walker y Avant en 2019, ajustada a 7 pasos para este estudio. La información se recolectó mediante búsqueda bibliográfica, en bases de datos SCOPUS, WEB OF SCIENCE, CINAHL, ProQuest, LILACS y búsqueda manual de referencias, sin límite de tiempo. Se incluyeron en el análisis 18 estudios originales y de revisión.

**Resultados:**

La clarificación conceptual define la ganancia del cuidado como una consecuencia positiva, percibida como enriquecedora, que emerge de la interacción entre componentes contextuales (individuales, relacionales, comunitarios y sociales), la calidad y cantidad de las actividades de cuidado directo, y la duración del rol de cuidador. Esta ganancia trasciende tanto el periodo activo de cuidado como la etapa posterior a su finalización.

**Discusión:**

La ganancia del cuidado contiene elementos contextuales, variables temporales y de carga de cuidado cuya interacción enriquece a la persona cuidadora, mejorando la experiencia y calidad del cuidado. Esta clarificación reconoce su naturaleza fenomenológica y facilitará su uso en la investigación y la práctica del cuidado en la demencia.

**Conclusiones:**

En el cuidado familiar en demencia, la ganancia del cuidado es una consecuencia enriquecedora para quien cuida y mejora la calidad del cuidado. Se sugiere considerar su naturaleza subjetiva e incluirla en estudios futuros y en la práctica de enfermería.

## Resumo

**Introdução:**

O conceito de ganhos do cuidado tem sido usado como sinônimo de aspectos positivos do cuidado, o que dificulta sua pesquisa e aplicação prática.

**Objetivo:**

Esclarecer o conceito de ganhos do cuidado no contexto da demência.

**Materiais e métodos:**

Esta análise conceitual utilizou a metodologia desenvolvida por Walker e Avant em 2019, adaptada em sete etapas para este estudo. Os dados foram coletados por meio de busca bibliográfica nas bases de dados SCOPUS, WEB OF SCIENCE, CINAHL, ProQuest e LILACS, bem como por meio de buscas manuais em referências bibliográficas, sem limite temporal. Dezoito estudos originais e revisões foram incluídos na análise.

**Resultados:**

O esclarecimento conceitual define os ganhos do cuidado como uma consequência positiva, percebida como enriquecedora, que emerge da interação entre componentes contextuais (individual, relacional, comunitário e social), a qualidade e a quantidade das atividades de cuidado direto e a duração do papel de cuidador. Esse benefício transcende tanto o período de cuidado ativo quanto a fase posterior ao seu término.

**Discussão:**

O benefício do cuidado abrange elementos contextuais, variáveis temporais e a sobrecarga do cuidador, cuja interação enriquece o cuidador, melhorando a experiência e a qualidade do cuidado. Este esclarecimento reconhece sua natureza fenomenológica e facilitará seu uso em pesquisas e na prática em cuidados com demência.

**Conclusões:**

No cuidado familiar de pessoas com demência, o benefício do cuidado é uma consequência enriquecedora para o cuidador e melhora a qualidade do cuidado. Sugere-se que sua natureza subjetiva seja considerada e incluída em estudos futuros e na prática de enfermagem.

## Introduction

Population aging and the increasing number of people affected by chronic diseases influence the prevalence of syndromes such as dementia, which affects more than 55 million people worldwide^1^,^2^. People living with dementia require special care, and this falls mainly on family members or close friends^3^-^6^.

A positive outcome of the caregiving experience is the care gain, which has been defined in the context of informal care as the extent to which the caregiver role is perceived to improve and enrich an individual's life space because of becoming a caregiver^7^. Gain is understood as benefits derived from the act of caring, and which extend beyond what can be psychological well-being. Recent studies have shown that informal caregivers experience a range of psychological and emotional benefits, including a sense of purpose, personal satisfaction, and strengthened family bonds^8^. These positive outcomes of caregiving can help caregivers better cope with the challenges and stresses associated with their role, significantly impacting their emotional well-being and quality of life^9^,^10^. Rewarding caregiving is considered to protect caregivers from adverse effects^11^ thus encouraging prolonged caregiver involvement despite feelings of burden or distress^12^,^13^.

The negative impact of providing unpaid care to a person with dementia has been widely documented, showing a decline in mental health, due to high levels of depression and anxiety, and physical health in the caregiver^14^,^15^ and social and economic difficulties^16^.

Caregiving gains have been studied primarily among caregivers of people living with dementia, from a stress and coping theory paradigm. Other authors have explored them from the perspective of positive psychology, finding, for example, that hope, as a strength, fosters a perception of gain in the caregiver's role. This can also be applied in caregiving contexts in other areas where long-term informal care is provided^17^. One study found that older caregivers who performed more medical or nursing care tasks reported greater gains, which were measured using a questionnaire with four statements related to confidence in one's own abilities, learning to cope with difficulties, connecting with the person being cared for, and feeling satisfaction in providing good care^18^. According to Kramer, gains are different from role satisfaction. The latter subtly indicates satisfaction (joy, pleasure) in having met one's own expectations and does not necessarily mean having obtained any benefit from the caregiving experience. On the other hand, there are the rewards of caregiving. Several studies have categorized the positive aspects of caregiving: benefits of a reward or gain for the caregiver, the care recipient, or both; and the nature of the satisfaction, whether interpersonal, intrapersonal, or outcome gain^19^. Consequently, the evidence shows that, to date, the definitions, characteristics, and uses of caregiving gain are not currently clear^20^.

For nursing, it is essential to recognize the benefits associated with the caregiver role as a relevant phenomenon, especially in contexts of high emotional demand such as caring for people with dementia. This recognition allows for a caregiving approach that goes beyond burnout, considering dimensions of personal growth, strengthened relationships, and existential meaning, given its relationship with favorable health outcomes for caregivers and the need for further research. However, the lack of conceptual clarity regarding these benefits and their indiscriminate use with what has been termed "positive aspects of care" hinders more precise research approaches, relevant interventions, and coherent care proposals contextualized to the real and potential needs of the dyads (the primary caregiver and the dependent person being cared for) and their families.

In this context, to facilitate understanding, it is necessary to analyze the concept of caregiving benefits. Concept analysis is a strategy that can be used to construct conceptual frameworks, theories, or research studies^21^-^23^, and can also be used as a methodological strategy to clarify, recognize, or define concepts that describe human phenomena, such as gains in care. The aim of this review is to clarify the concept of gains in care in the context of dementia.

## Materials and Methods

The analysis of the concept of care gains was conducted according to the Walker and Avant methodology (2019), which consists of eight steps. However, for this study, it was adapted to seven steps by merging steps 5 and 6^24^. The first step was to select the concept of "care gains," and the second was to establish the purpose and objective of this analysis. The third step consisted of identifying and clarifying the use of the concept. In the fourth step, the attributes (characteristics) of care gains were identified, and the articles and documents found were analyzed rigorously and intensively to clarify the concept. In the fifth and sixth steps, only the model case was presented, as it accurately illustrates the essential attributes of the concept. Furthermore, the model case may contain elements typical of the opposing cases in step six, since the negative aspects of the caregiving experience coexist with the positive ones in this case of care gains. This coexistence reflects the conceptual multidimensionality of the phenomenon, in which affective, ethical, relational, and contextual dimensions intertwine, lending depth and complexity to its theoretical delimitation. A real-life case study is presented, focusing on a caregiver and their family member with dementia who live in the community. In the sixth step, we address the antecedents, which are the actions or conditions necessary for the concept to occur; the consequences, which are the results of the event; and the elements that could influence the presence of caregiving gains. In the seventh step, the empirical references found were described. For steps 3 through 7 A targeted literature search was conducted in databases, for studies, books, guides, official documents and others, which allowed the identification of similar concepts useful for clarifying the selected concept^24^,^25^. In this way, certain conceptual elements are identified, and the chosen concept is distinguished from those found during the previous review, as well as possible empirical referents.

**Data sources**

The information used was collected through a bibliographic search in the databases SCOPUS, WEB OF SCIENCE, CINAHL, ProQuest, and LILACS, and a manual search of reference lists of retrieved articles. The search strategy included in all cases ((caregiving gain)) OR (caregiving benefits) OR (caregiving reward) OR (positive caregiving outcomes) AND (caregivers) OR (carer)) OR (Family Caregivers) AND ("dementia") OR ("dementias") OR ("Alzheimer disease")). Regarding inclusion criteria, original and review articles were selected that included the definition of care gain and/or the “Uses of the Concept,” “Attributes,” “Background,” “Consequences,” and “Empirical References” in samples of caregivers of people with dementia, filtered by language (Spanish, English, Portuguese) with no time limit. Editorials, letters to the editor, abstracts, and expert opinions were excluded. Using Rayyan AI software, duplicate files were excluded, and 131 articles were read in full text. Finally, 18 articles were included, and all collected data are freely available for consultation on Mendeley Data^26^.

**Figure 1:**
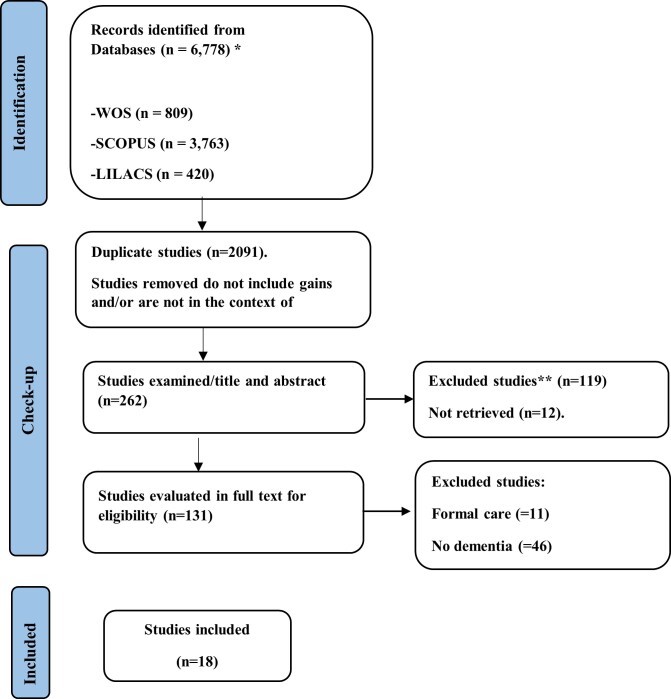
Flowchart of targeted literature review

*Flowchart of targeted literature review*

## Results

**Concept selection**

The selected concept is the “caring gain”, defined as the extent to which *the caregiver role is perceived to improve and enrich an individual's living space, including affective and practical benefits associated with caregiving tasks*.

**Purpose of Concept Analysis**

The relevance of describing and clarifying the concept of care gains in dementia contexts lies in the need to value its various elements (personal growth, improved relationships, and spiritual growth), which are still unknown to many care professionals who work with caregivers of people with dementia. Identifying these elements is crucial for promoting and facilitating care interventions and support that are consistent with their needs, and for using it as a conceptual framework for both research on the positive experience of informal care in dementia and direct care for caregivers.

**Clarification and use of the concept**

This concept of profit has been defined in various ways in literature. For example, according to the dictionary of the Royal Spanish Academy, profit is defined as: the action and effect of winning, or the utility that results from dealing, commerce or other action, and as synonyms (benefit, fruit, yield, profit, advantage, product)^27^.

In scientific research, different terms have been used. For example, the term “care satisfaction” has been defined as an important dimension of care evaluation, understood as the benefits that accumulate for the caregiver through their own efforts^28^, “subjectively perceived gains from desirable aspects of, or positive affective returns from, caregiving”^29^, satisfaction can be associated with caregiving experiences that give a positive focus to life^30^,^31^. It has also been defined as the degree of perceived higher self-esteem derived from caregiving, as a positive dimension for evaluating care^32^,^33^. This satisfaction is measured by the positive perception of specific outcomes of caregiving , expressed as closeness with the person being cared for, feeling appreciated, increased self-esteem, and recognition of one's own caregiving abilities. Furthermore, it has been suggested that satisfaction is not related to stressors, but it does determine positive affect for spouses and adult children; high levels of effort result in greater burden and satisfaction^28^.

From the existentialist theoretical perspective that analyzes human existence in a universe that can be hostile or indifferent and, moreover, inexplicable, placing a strong emphasis on the freedom to make decisions, as well as on the responsibility and repercussions of said decisions, the gain from caregiving is understood as personal growth and finding meaning in the process of caring; thus, in the case of caregivers of people with dementia, They created meaning in the experience of caring by making decisions to use a positive attitude and sense of humor when responding to life's adversity^34^. For Pearlin et al.^35^ in 1990, caring gain is the measure of personal enrichment expressed as inner growth achieved by people when facing serious challenges assumed by caring for other people.

On the other hand, within the framework of Lazarus and Folkman's Stress Appraisal Theory (1984), as cited in Wallsten and Snyder^36^, they have defined gain as elevations or stimuli that mean feeling well-being, happiness and joy derived from daily care.

Recently, other studies defined gain as benefits of care: (confidence in their abilities in difficult situations, satisfaction with the outcome of care and greater closeness to the person being cared for)^37^, self-gains or greater benefits of care^38^. Other authors consider that they are experiences related to strengths or benefits beyond the capacity to provide care, which therefore contains metacognitive elements that allow them to reflect on their lives, relationships, and on their acts of care^39^, sas well as a humanity of the caregiver understood from love and devotion, highlighting the inherent value of the dyadic relationship and not from egocentrism or instrumentalism^40^. On the other hand, from the perspective of Ryff's elements of psychological well-being, the gain from care refers to personal benefits of care, identifying that variables such as depressive symptoms, subjective strain, duration of care, daily care time, physical complaints, and a good quality of relationship between the caregiver and the person with dementia are significantly associated with these benefits of care^41^.

Gain also refers to the perceived rewards of the caregiving role^42^,^43^. A recent study showed that significant predictors of well-rewarded family caregivers were being an adult child of the care recipient, having a high perceived level of mutuality, and a high perceived level of preparedness. This suggests that promoting the perception of reciprocity and capabilities in caregivers enhances the perception of reward in the caregiving role in the context of dementia. Similarly, it has also been found that some caregiver characteristics, such as age, gender, relationship to the care recipient, and social support, influence the perception of greater gratification^12^.

Broadly speaking, the concept of care gain was defined in 1997 as the extent to which the caregiving role is perceived to improve and enrich an individual's living space because of becoming a caretaker and may include any positive, effective or practical gain experienced as a direct result of being a caretaker. In line with the above, Sanders^43^ in a qualitative study with caregivers of family members with Alzheimer's disease, noted three themes related to caregiving gains: (a) spiritual growth and faith, (b) personal growth (changes in the caregivers' lives, greater patience, care, and gratitude for the opportunity to care for someone); and (c) feelings of mastery based on personal achievements.

Furthermore,, Netto et al.^44^ 2009, in a qualitative study, defines caregiving gains as advances experienced by family caregivers of people with dementia, which involve: personal growth in terms of cultivating deeper patience and understanding, becoming stronger and more resilient, increasing self-awareness, and gaining more knowledge for caregiving; gains in relationships with care recipients; increased family cohesion for mutual support; or in their ability to interact with other older people; and higher-level gains in terms of positive changes in life philosophy (e.g., less emphasis on material things and money), spiritual growth, and altruism.

Regarding factors that influence the perception of gain, it has been identified that some sociodemographic aspects of the caregiver, such as gender, caregiving time, and the number of caregiving activities, influence the former. In this sense, a recent study revealed that gender plays a differential role in the perception of gain; being greater in men than in women. This could be because female caregivers experience greater psychological distress, which can alter the perception of gain from caregiving^45^.

In this context, it is important to point out that the gain from care becomes a particularly positive reference point related to the caregiving function, and forms part of a set of beneficial aspects that arise from the experience of caring. Therefore, it should not be understood solely as the satisfaction of the caregiving role, since it encompasses the complexity of the positive experience and involves human dimensions beyond positive emotions.

**Determining the attributes of the concept**

Based on the analysis in the literature, the three main attributes of caregiving have been determined, which are dynamically interrelated with each other and with other external factors, shaping the positive experience of caring.

**Figure 2:**
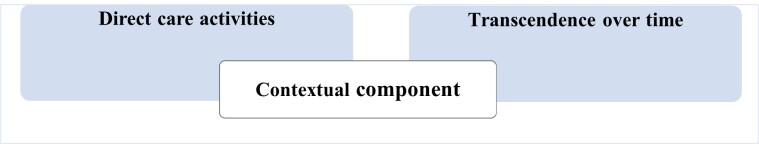
Attributes of the gain from care

**Contextual components**

***Individual components:*** These relate to the caregiver's personal progress and achievements in caregiving skills, identifying strengths and weaknesses, motivations and interests, and sociodemographic variables (age, gender, relationship to the person being cared for). Regarding the above, caregivers report a sense of personal growth by feeling altruistic, with a stronger spiritual or religious belief, and increased faith in a higher power^34^,^43^. Other research has indicated that family caregivers who provide care driven by love and reciprocity rather than external obligation tend to discover greater meaning in the caregiving experience, leading to a higher level of gain from caregiving^46^. On the other hand, according to Ponsoda in 2024, Gain in caregivers has been positively associated with lower educational attainment, older age, as well as low perceived burden and good mental health; it was also positively associated with the caregiver's sense of competence and with the use of encouragement and active management strategies^45^. They also found that higher gain scores were obtained by caregivers who were unemployed, had minimal financial hardship, and attended educational and support programs for caregivers.

***Relational components:*** This refers to family relationships, trust in home care, and life adjustments. It is important to note that the quality of the dyadic relationship influences the perceived benefits of caregiving, given the social interdependence that modifies the caregiving situation and the constant changes associated with the progression of the care recipient's decline. Therefore, having a good relationship with the care recipient, both in the past and present, has been identified as an important predictor of positive caregiving experiences among family caregivers of people with dementia^47^. It has also been described that emotional and interactional social support offered family caregivers a constant source of inspiration, peace of mind, mutual encouragement, and social recognition as they adapted to the demands of family care^47^.

***Community components:*** This refers to the availability and access to resources that influence the caregiving experience, such as health services and social and educational support available to caregivers. Thus, one study reported that caregivers who participated in psychoeducational programs expressed improvements in competence and personal benefit that were maintained at 12 months^48^.

***Social components:*** This refers to cultural and ethnic aspects as factors influencing the perception of benefits. Culture and religion provide a kind of intrinsic motivation. It is also noted that caregivers accepted Alzheimer's disease because it comes from God and provides their obligation to care in the right way, thanking God for preserving the mental and physical well-being of their family member during the progression of the disease^49^. Racial minority caregivers have been less studied; however, ethnic/racial aspects can significantly influence the perception of benefits of caregiving. Thus, a study with African American and Hispanic caregivers indicated that caregiving experiences are shaped by individual and interpersonal factors, with African Americans being more affected by age, duration of caregiving, and social support (individual and intrapersonal), and Hispanic Americans by factors such as being married and access to transportation (intrapersonal and organizational)^50^. Another study indicates that Korean-American caregivers were more influenced by the social support they perceived given the cultural principle of filial piety, which states that children are responsible for caring for their elderly parents, or that daughters and daughters-in-law should care for people with dementia regardless of their personal relationship with them^51^.

**Transcendence over time**

Remaining in the caregiver role increases the likelihood of finding greater meaning and gain from caregiving and of continuing to do so even after caregiving is discontinued. This aligns with a study that identified higher gain scores among individuals who had been the primary caregiver for more than three years and who spent at least 60% of their time each week on caregiving tasks. Furthermore, the perception of gains has been shown to be stable over time, as revealed by a multi-pathway study conducted over two years and up to six months after the death of the older person being cared for. This study found that while negative caregiving experiences varied more widely among caregivers, positive caregiver experiences remained high^52^. The persistence and frequency of long-term caregiving for a family member with dementia may be linked to intense attachment, identification, and commitment to the well-being of the person being cared for, and to a unilateral stance observed in numerous family caregiving relationships^40^.

**Direct care activities**

Direct caregiving activities are stressful due to the time and knowledge demands they require, but they can also provide opportunities to learn new caregiving skills, implement symptom management strategies, and effectively manage available resources for assisting the person with dementia. In this regard, the literature reports that caregivers with a more advanced stage of dementia had higher scores on the gain assessment. Furthermore, it is also evident that medical or nursing tasks make caregivers feel they are directly benefiting their partners, which further enhances their perception of gains^53^. Routine and spontaneous caregiving activities resulted in greater feelings of intimacy between the caregiver and the person with dementia, improving the quality-of-care delivery and contributing to the personal enrichment process^19^.

**Model case**

Mary is the daughter of Luisa, 80, and Juan, 89. She has lived with them for over 12 years because, although she married and had children, she never distanced herself from her parents and maintained a close, supportive, and caring relationship with them (relational contextual component). Eight years ago, her father suffered a stroke that left him with severe disabilities, confining him to bed in a state of severe dependency. He has difficulty communicating due to severe mixed hearing loss, dysarthria, and aphasia. He also requires assistance with all basic and instrumental activities of daily life, and Mary has been his primary caregiver ever since. Her father's condition requires Mary to implement various forms of communication, reposition him every two hours to prevent pressure injuries, administer prescribed medications, assist with feeding, manage his healthcare, and take him to necessary medical appointments, among other activities (direct care activities). Mary's care routine for her father is continuous and extends 24 hours a day. She placed a bed in the same room to feel closer to him and so he would always know she was available to help. Juan is increasingly exhausted, wanting only to sleep, and has begun to develop redness in the sacral area and right ankle, a situation that worries Mary because she knows it's another complication that represents a further decline in her father's health. She says that while she sometimes feels overwhelmed by the burden of caregiving, she doesn't consider giving it up because she loves her parents and it's her responsibility as a daughter to "pay it forward." She also feels she has learned to do it very well. Her sister lives nearby and supports her in some decisions regarding her father and sometimes when she must go to the market, the doctor, caregiver training sessions, and meetings of the support group she recently joined. She has requested assistance from the Health and Social Services Center, and they have sent a nurse to guide her on the prevention and management of pressure injuries. In addition, she has secured the support of a caregiver who primarily assists with household chores three days a week and whom she is training to also occasionally assist her father (community component), given that she does not consider institutionalizing her father (long-term impact). Mary lives with her husband and parents, receives no income, and therefore depends on her husband's pension and support. She has a spinal (lumbar) injury that has caused her chronic pain for over six years, which, she says, sometimes limits her mobility, strength, and walking, complicating her ability to care for her father. She considers herself an active and committed caregiver but also acknowledges that her health is not the best and that she has had to postpone aspects of her own life and interests. Despite this, she says that the entire caregiving process has made her more diligent, capable, and skilled at assisting them, changing diapers, bathing, feeding, and creating entertainment strategies to share with her father, since she cannot even take him out of his room. Mary states that she is satisfied taking care of him, seeing her father protected at home, although he cannot speak or move, it is enough for her that he listens to her, or looks at her when she speaks to him, and she expresses that it makes her happy to be the caregiver of her parents ( individual contextual component ).

**Background and consequences**

**Figure 3:**
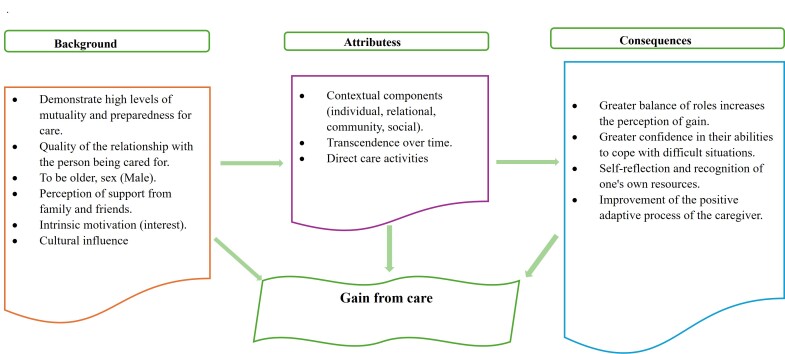
Conceptual Model of care gain

The following describes the background and consequences that contribute to caregiving gains. The experience of informal caregivers in dementia is unique, involving a complex process of multiple interactions between contextual factors, caregiving, and time that shape their perception of gains associated with the caregiving role Figure 3.

The perceived gain has relevant antecedents present in the caregiver such as presenting high levels of mutuality and preparedness for care, quality of the relationship with the person being cared for, being older, being male, although women also perceive gain from the care role and having perceived social support from family and friends mainly, as well as remaining longer and with more care activities^19^,^45^,^48^. Finally, having intrinsic motivation to care and the influence of cultural factors mainly in women^54^. While most caregivers worldwide are women, this overrepresentation does not imply that femininity itself facilitates the perception of benefits in caregiving, but rather that it modulates it. On the other hand, these factors generate consequences such as greater role balance, which increases the perception of gains; greater confidence in their abilities to cope with difficult situations; self-reflection; and self-recognition, all of which favor a positive adaptive process^38^. A harmonious dyadic relationship increases the caregiver's motivation by fostering feelings of accomplishment and meaning in their role that persist beyond the active caregiving period^19^.

**Empirical determinants**

Various scales have been used to measure caregiving gains from a multidimensional perspective and in diverse caregiving contexts. These include the Finding Meaning Through Caregiving Scale (FMTCS), the Experience of Caregiving Inventory (ECI), and the Positive Experiences Scale (PES), which allow for exploring the subjective and relational impact of caregiving^55^. Furthermore, caregiving gains have been measured from the conceptualization of caregiver role satisfaction, and more recently, instruments developed using the positive aspects model have been employed. In dementia care contexts, the most validated and widely used instrument, even as a generic measure of positive aspects of caregiving, is the Gain in Alzheimer's Care Instrument. The first four items refer to personal gain, the next three to relationship gain, and the final three to higher-level gain. It is a 10-item Likert scale with a score range of 0 to 40, where 0 (strongly disagree) to 4 (strongly agree). Higher scores indicate greater gain^56^.

The Positive Aspects Scale (PAC) has nine items to measure the positive aspects of caring for people with dementia. It was developed from the Lawton Caregiver Satisfaction Scale, which comprises two factors: “self-affirmation” and “positive outlook.” Each item is rated on a 5-point ordinal scale ranging from 1 (strongly disagree) to 5 (strongly agree), widely used to measure gains and positive aspects of family caregiving. Since caregiving gains are a multidimensional concept, further analysis of its component attributes is needed to consider aspects such as gender, social support received and perceived, and the duration of caregiving, which significantly influence the perception of gains and have not yet been included in existing measures.

**Operational definition of the concept**

Considering the above, the gains from caregiving can be defined as the positive, enriching outcome that emerges from the interaction between contextual components (individual, relational, community, and social), the quality and quantity of direct care activities, and the length of time spent in the caregiving role. These gains extend beyond both the active caregiving period and the time following its termination.

## Discussion

Research on the positive outcomes of caregiving has highlighted the "caring gain" as a concept that benefits caregivers both emotionally and practically, improving their psychological well-being and the quality of care they provide to people with dementia. This concept has been explored primarily within the framework of stress and coping theory, from positive psychology and existentialism, and based on Kramer's 1993 Caregiver Adaptation Model, as cited in Lawton et al^31^.

The benefits derived from caregiving are related to sociodemographic factors such as gender. While men report slightly higher levels of benefits than women^44^, one possible explanation is that, from a masculine perspective, men consider advancement in the caregiving role a significant achievement, unlike women, who perceive the caregiving role as socially imposed. Furthermore, according to the literature, women experience more psychological distress, which diminishes their perceived benefits. In this regard, another study indicated that, compared to male caregivers, female caregivers had a greater caregiving burden, with 90% of them acting out of moral obligation, reciprocity, and love, and relying more on religion for comfort. This study also found that female caregivers used fewer protective coping mechanisms^57^. Therefore, the female experience of caregiving in dementia contexts is shaped by a logic marked by structural, affective, and moral vulnerabilities, which hinders equitable access to the subjective benefits of caregiving, reflecting that femininity is not a conclusive attribute that facilitates the gain of caregiving, but rather a condition that influences how it is perceived.

The time factor represents a fundamental axis in the process of perceiving gain, since greater permanence in the role and a greater number of hours dedicated to care increase the probability of experiencing gain^58^. However, this attribute has not been integrated into existing methods of measuring gain.

Finally, the caregiving activities themselves, considering both quality and quantity, constitute the element that generates the greatest burden, but also the greatest closeness with the person being cared for. Furthermore, the level of complexity of care demands knowledge and new learning every day, transforming daily routines into complex and challenging activities that can be stressful but once overcome, produce feelings of accomplishment and mastery in the caregiver^19^.

It is noted that the subjective nature of interpretations for understanding the gain of care should be considered for future studies and in care practice, so that actions in favor are contextualized in the reality of those who experience it.

## Conclusions

Caregiving gains are positive outcomes reported in the experience of caring for people with dementia. These gains can be emotional, cognitive, behavioral, or interpersonal. This conceptual clarification found that caregiving gains result from the interaction of various contextual elements, including direct care activities influenced by caregiving time, which extend beyond the permanence of the caregiving role. This constitutes a conceptual contribution to nursing theories focused on caregiver adaptation processes, the formulation of care plans contextualized to the real needs of caregivers, and training focused on promoting positive caregiving outcomes as a paradigm shift.
